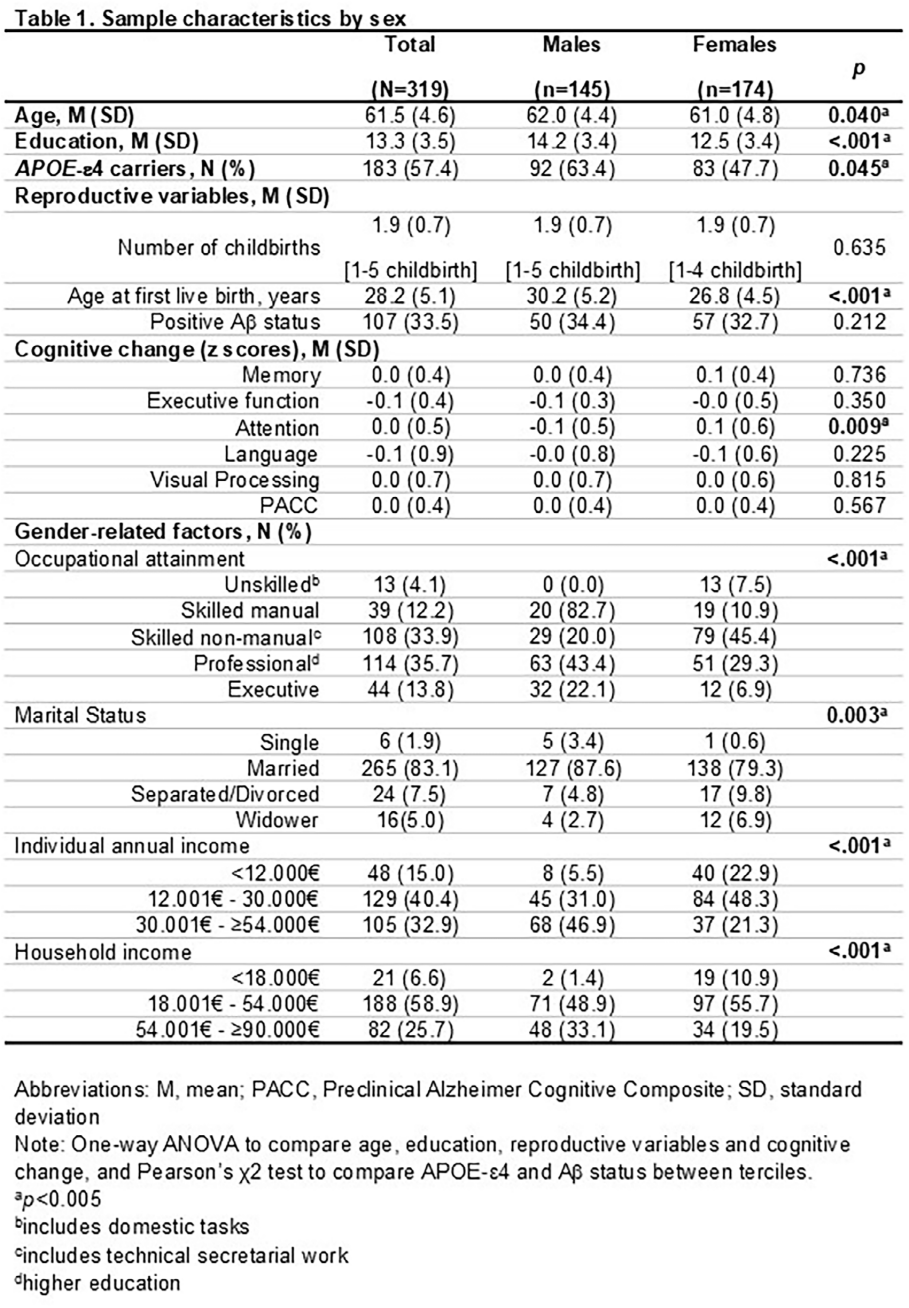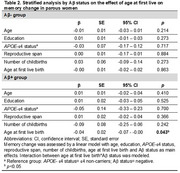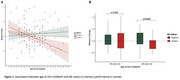# Late motherhood linked to accelerated memory decline in amyloid‐positive cognitively unimpaired women

**DOI:** 10.1002/alz70856_101674

**Published:** 2025-12-24

**Authors:** Anna Brugulat‐Serrat, Clara Gallay, Gonzalo Sánchez‐Benavides, David López‐Martos, Ana Fernández‐Arcos, Henrik Zetterberg, Kaj Blennow, Marc Suárez‐Calvet, Oriol Grau‐Rivera, Juan Domingo Gispert, Erin E. Sundermann, for the ALFA Study

**Affiliations:** ^1^ Barcelonaβeta Brain Research Center (BBRC), Pasqual Maragall Foundation, Barcelona, Spain; ^2^ University Pompeu Fabra, Barcelona, Barcelona, Spain; ^3^ Centro de Investigación Biomédica en Red de Fragilidad y Envejecimiento Saludable (CIBERFES), 28089, Madrid, Spain; ^4^ IMIM (Hospital del Mar Medical Research Institute), Barcelona, Spain; ^5^ Barcelona beta Brain Research Center (BBRC), Pasqual Maragall Foundation, Barcelona, Spain; ^6^ Hospital del Mar Research Institute, Barcelona, Spain; ^7^ UK Dementia Research Institute at UCL, London, United Kingdom; ^8^ Department of Neurodegenerative Disease, UCL Queen Square Institute of Neurology, University College London, London, United Kingdom; ^9^ Department of Psychiatry and Neurochemistry, Institute of Neuroscience & Physiology, the Sahlgrenska Academy at the University of Gothenburg, Mölndal, Gothenburg, Sweden; ^10^ Laboratory of Clinical Chemistry, Sahlgrenska University Hospital, Gothenburg, Sweden Institute of Neuroscience and Physiology, the Sahlgrenska Academy at the University of Gothenburg, Mölndal, Sweden; ^11^ Department of Psychiatry and Neurochemistry, Institute of Neuroscience and Physiology, The Sahlgrenska Academy at the University of Gothenburg, Mölndal, Västra Götalands län, Sweden; ^12^ Sahlgrenska University Hospital, Clinical Neurochemistry Laboratory, Mölndal, Sweden; ^13^ Servei de Neurologia, Hospital del Mar, Barcelona, Spain; ^14^ BarcelonaBeta Brain Research Center (BBRC), Pasqual Maragall Foundation, Barcelona, Spain; ^15^ Centro de Investigación Biomédica en Red de Fragilidad y Envejecimiento Saludable (CIBERFES), Instituto de Salud Carlos III, Barcelona, Spain; ^16^ CIBER Bioingeniería, Biomateriales y Nanomedicina (CIBER‐BBN), Madrid, Spain; ^17^ University of California, San Diego, La Jolla, CA, USA

## Abstract

**Background:**

Both nulliparity and multiparity have been linked to an increased risk of dementia. However, evidence is inconclusive and requires further study. To further investigate the link between pregnancy history and cognition, we explored the link between age at first childbirth and cognitive change in cognitively unimpaired (CU) women and men at risk of AD.

**Method:**

We included 319 CU individuals at baseline (mean age 61.5 [4.6] years, *n* = 174 [54.5%] women) with biological children from the ALFA+ cohort. Age at first childbirth was defined as continuous and dichotomic (<29 vs ≥29 years). Amyloid (Aβ) positivity was defined as CSF Aβ42/40 ratio<0.071. Cognitive change (3‐year follow‐up) was measured with the PACC and domain‐specific composites. Multivariable regression models predicting cognitive change were adjusted for age, education, *APOE*‐ε4, reproductive span, and number of childbirths. Interaction terms among sex, age at first childbirth, and Aβ status were modeled. A stratified analysis by Aβ status in women indetified the age threshold at which Aβ moderates the association. We tested whether socioeconomic factors (e.g., income, occupational attainment) mediate associations.

**Result:**

Women were younger at first childbirth than men (*p* = 0.040, Table 1). Sex interacted with age at first childbirth and with Aβ status to predict a change in memory performance only (*p* = 0.014). Specifically, only Aβ+ women showed a negative association between age at first childbirth and memory change over time (Figure 1A), with a significant effect observed from age 29 onwards. Aβ+ women with first birth at ≥29 years old show a 52.4% steeper memory decline compared to Aβ‐ women in the same first childbirth age group (Figure 1B). Socioeconomic factors did not significantly mediate the relationship in women. Paternal age at first childbirth or its interaction with Aβ did not relate to any cognitive change in men.

**Conclusion:**

In women with preclinical AD (Aβ+/CU), late motherhood was associated with a more deleterious effect on the change of memory performance later in life. Findings suggest a complex interplay between pregnancy history and AD pathology in women. The lack of findings in men and influence of socioeconomic factors suggest that pregnancy‐related biological or hormonal factors may be driving the relationship in women.